# Different Macrophage Type Triggering as Target of the Action of Biologically Active Substances from Marine Invertebrates

**DOI:** 10.3390/md18010037

**Published:** 2020-01-02

**Authors:** Lyudmila S. Dolmatova, Igor Yu. Dolmatov

**Affiliations:** 1 V.I. Il‘ichev Pacific Oceanological Institute, Far Eastern Branch, Russian Academy of Sciences, Baltiyskaya 43, 690041 Vladivostok, Russia; 2 National Scientific Center of Marine Biology, Far Eastern Branch, Russian Academy of Sciences, Palchevsky 17, 690041 Vladivostok, Russia; idolmatov@mail.ru

**Keywords:** marine compounds, inflammation, immunostimulation, macrophage polarization, cytokines, NO, ROS

## Abstract

Macrophages play a fundamental role in the immune system. Depending on the microenvironment stimuli, macrophages can acquire distinct phenotypes characterized with different sets of the markers of their functional activities. Polarization of macrophages towards M1 type (classical activation) is involved in inflammation and the related progression of diseases, while, in contrast, alternatively activated M2 macrophages are associated with the anti-inflammatory mechanisms. Reprogramming macrophages to switch their phenotypes could provide a new therapeutic strategy, and targeting the M1/M2 macrophage balance is a promising current trend in pharmacology. Marine invertebrates are a vast source of the variety of structurally diverse compounds with potent pharmacological activities. For years, a large number of studies concerning the immunomodulatory properties of the marine substances have been run with using some intracellular markers of immune stimulation or suppression irrespective of the possible application of marine compounds in reprogramming of macrophage activation, and only few reports clearly demonstrated the macrophage-polarizing activities of some marine compounds during the last decade. In this review, the data on the immunomodulating effects of the extracts and pure compounds of a variety of chemical structure from species of different classes of marine invertebrates are described with focus on their potential in shifting M1/M2 macrophage balance towards M1 or M2 phenotype.

## 1. Introduction

Macrophages play a key role in initiating and maintaining the inflammatory response, as well as in tissue repair. Inflammation is a complex mechanism involved in the defense of the organism against pathogens. However, if unchecked, inflammation will result in cellular and tissue damage that can lead to chronic disease [1]. Thus, in response to harmful stimuli, including bacterial lipopolysaccharide (LPS), macrophages produce high levels of pro-inflammatory cytokines such as tumor necrosis factor (TNF)-α and interleukins (IL)-6, IL-8, IL-1β [2]. This results in activation of the inhibitory subunit of nuclear factor kappa B (NF-κB), inhibitor of kappa B (IκB)- kinase, followed by the phosphorylation of IκB protein bound to NF-κB. Afterwards, NF-κB translocates to the nucleus, where it binds to the promoter region of several genes. NF-κB is involved in regulation of expression of several pro-inflammatory enzymes like cyclooxygenase 2 (COX-2) and inducible nitric oxide synthase (iNOS). iNOS is responsible for synthesis of nitric oxide (NO), which is found in cells in large amounts during inflammation. In turn, COX-2 and phospholipase A2 (PLA2) induce a production of prostaglandin E2 (PGE2) [3]. The significant changes in the amount/activity of those molecules allow to use them for monitoring inflammation, and evaluation of the effects of new compounds on those markers in vitro is usually used for exploring their potential inflammatory properties. Additionally, in recent years, stress that arises from accumulation of unfolded proteins within a cell’s endoplasmic reticulum (ER) was identified, and ER stress was found to participate in LPS-induced immune response and regulate inflammation in some pathological models [4]. Therefore, some markers of ER stress, e.g., a transcription factor C/EBP homologous protein (CHOP) [5], are sometimes evaluated in the screening compounds for immunomodulating activity.

About four decades ago, the macrophages were first shown to be a morphotypically heterogeneous cell population while exposed to different cytokines [6,7]. Later, these cells were shown to have ability to undergo activation to pro-inflammatory M1 (classically activated) or anti-inflammatory M2 (alternatively activated) phenotypes in response to different exogenic and endogenic stimuli [8]. Among those stimuli, interferon-gamma (IFN-γ) combined with LPS, and mediators of ER-stress activation, such as pancreatic EIF-2alpha kinase, can be mentioned as drivers of the M1 phenotype [9]. Synthetic glucocorticoid dexamethasone and IL-4, IL-10, and IL-13 [8] are inducers of M2 activation. In turn, M1 macrophages release proinflammatory cytokines, such as IL-1β, IL-6, IL-12, IL-18, IL-23, TNFα, type I IFN, and numerous chemokines. Moreover, these cells produce high levels of NO and reactive oxygen species (ROS) [10]. Phenotypic characteristics for M1 macrophages are CD64 and CD80 [11], and also CD14 but not CD16 expression [8]. M2 cells display a high activity of arginase-1 and secrete high levels of IL-10, IL-13 [10,11], and IL-8 [12]. M2-like macrophage phenotype is identified based on the expression of CD64 and CD209 [9].

Moreover, two types of macrophages use different metabolic pathways for energy generation: glycolysis in M1 type [13], and oxidative metabolism in M2 cells [14]. In addition, M1 type has a higher ratio of reduced-to-oxidized glutathione (GSH/GSSG) compared to that in M2 type, and GSH depletion can switch macrophages from the M1 towards the M2 phenotype [15]. The diversity of the microenvironment results in a spectrum of in vivo macrophage phenotypes and functions so that the classical and alternative ways of polarization of macrophages are considered to lead to the extremes of the spectrum of possible phenotypes [16]. Additionally, a new type of macrophages was recently described—regulatory macrophages (MRs). They play a crucial role in anti-inflammatory restriction of inflammation during innate and adaptive immune responses, and dehydrogenase/reductase 9 (DHRS9) is suggested to be their marker [17].

A high plasticity of macrophages and the fact that mixed features of the two types are sometimes present in macrophage populations [18] put a question whether two clearly distinct types of macrophages are present in vivo. However, Jablonski et al. [17] identified a new set of common and distinct M1 and M2 genes regulating pathways linked to M1 and/or M2 phenotypes. This indicates the presence of really distinct traits of the two macrophage types. Moreover, in invertebrates, the expression of arginase in macrophages after stimulation with different stimuli was shown to occur without participation of characteristic for vertebrates Th2 cytokines IL-4/IL-13, and this indicates that the protein regulation of wound healing in lower animals is produced by innate immunity cells [19]. In addition, recent studies showed the existence of two types of macrophage—like phagocytes (referred to as P1 and P2), separated by the gradient centrifugation, in holothurians (Echinodermata). The P1 type has a high level of NO as a marker, increased levels of GSH, and reactive oxygen species (ROS) compared to those in P2, but P2-type had increased arginase activity compared to that in P1 [20,21]. The two cell types also differ in their surface receptor expression [17], but under some stimuli both types could acquire the features of each other [21]. These data indicate the appearance of distinct types of macrophages already in evolutionally ancient animals like echinoderms.

The known intracellular signaling ways and mechanisms of macrophage transformation via classical or alternative ways as well as their involvement in pathogenesis of many diseases are more fully described in several reviews [9,22,23]. It was clearly shown that development of a number of diseases is resulted from the M1/M2 disbalance. However, macrophage polarization may be reprogrammed, and a switching from M1 to M2 type was shown to be one of the mechanisms of action of well-known drugs and is considered to become a target for newly developed pharmacological agents [24,25]. Thereby, a number of natural compounds of plant origin were described to be potent for macrophage reprogramming in the last decade [25], but little is known about such substances from marine hydrobionts, in particular, invertebrates. A vast number of marine bioactive compounds were discovered in the last five decades [26,27], and their number is increasing with the development of new methods and instruments of the ocean research and new discoveries especially due to deep-sea studies during the last few years [28]. For years, a number of crude extracts and pure compounds were tested for bioactivities, first of all, for their presumable anti-inflammatory properties, using the markers of inflammation. Only few reports emphasizing the influence of the marine substances on macrophage phenotypes have appeared in the last decade. Nevertheless, taking into consideration, that some intracellular markers of inflammation characterize also the macrophage phenotypes, the results obtained with using such markers may indicate macrophage-polarizing potential of substances tested. Noteworthy, the most studies were conducted in vitro, and LPS was used usually to induce signaling in primary macrophages or cultured cell lines. Recent studies indicate that effects of LPS are time- and concentration-dependent [29,30]. Thus, in bone marrow-derived macrophages short-time (several hours) incubation with 0.1 μg/mL LPS inhibited M1 polarization, while long-term (24 h) incubation promoted M1 and inhibited M2 polarization [30]. Therefore, the results obtained in different cell lines and under different incubation conditions may be distinct, and attention has to be paid to this fact. In this review, the known data on the effects of the extracts and substances from some groups of marine invertebrates (Cnidaria, Porifera, Crustacea, Mollusca, Echinodermata) on the expression of inflammatory and macrophage phenotypic markers are summarized and analyzed with an accent on the promising compounds for M1–M2 macrophage transition targeting.

## 2. Natural Compounds with Macrophage Phenotype Modulating Activities from Marine Invertebrates of Different Taxa

### 2.1. Cnidaria

Phylum Cnidaria contains over 11,000 species [31] and is one of the richest in secondary metabolites of various structure phyla [32]. These species remain an untapped source; however, a great variety of chemically unique structures with promising bioactive properties was described for this phylum until now [32].

#### 2.1.1. Soft Corals

Soft corals (Alcyonacea) is an order of cnidarians belonging to class Anthozoa, subclass Octocorallia. They have proven to be rich sources of several groups of chemically different compounds with anti-inflammatory properties, with mechanisms of action that are reviewed in a number of reports [32,33]. Anti-inflammatory effects of compounds isolated from Taiwanese corals from 2008 until 2012 were fully described in review of Wei et al. [33], where the authors presented all chemical structures of compounds and described their anti-inflammatory activities studied in different immune cells (neutrophils and macrophages) in vitro and in animal models of inflammation. Presenting here some data reported in the review of Wei et al. [33], we focus only on those concerning the capacity of natural substances from the soft corals to inhibit LPS-induced upregulation of NO- and eicosanoid-generating enzymes iNOS and COX-2, respectively, and also the level of NO and TNF-α which are considered to be markers of both inflammation [6,11] and macrophage classical activation pathway [11,20,34,35] in RAW264.7 cells. Far from being a common character for potential anti-inflammatory compounds, of 9 triquinane-type sesquiterpenoids from soft corals *Capnella imbricata* described in the work of Wei et al. [33], only three were shown to inhibit expression of both iNOS and COX-2 proteins in LPS-stimulated cells and one more—inhibits only iNOS protein [36]. Of three nardosinane-type sesquiterpenoids from *Paralemnalia thyrsoides* [37], none influenced these markers; and of four nardosinane-type sesquiterpenoid flavalins from *Lemnalia flava* only one inhibited expression of iNOS and COX-2 proteins [38]. Of six aromadendrane-type sesquiterpenoid lochmolins from the soft coral *Sinularia lochmodes*, none were able to inhibit accumulation of iNOS protein, and only four compounds inhibited COX-2 (at concentrations of 10–100 μM) [39]. Only one of four selinane- and oppositane-type sesquiterpenoids from soft coral *Sinularia leptoclados* inhibited iNOS protein expression [40], and one of three ylangene-type sesquiterpenoids from *Lemnalia flava* reduced iNOS and COX-2 protein accumulation [38]. Inhibition of iNOS expression was described also for sesquiterpenoids erectathiol from *Nephthea erecta* [41] and for scabralin A from *Sinularia scabra* [42].

The most anti-inflammatory active were cembrane-based diterpenoids. Almost all of them, described in the review of Wei et al. [33], were found to have iNOS and COX-2 inhibiting ability: gibberosene B from *Sinularia gibberosa* [43], durumolides A, C [44], F and K [45], 13S-hydroxylobolide [44], deacetyl-13-hydroxylobolide, (7*E*,11*E*)-13,18-dihydroxy-3,4-epoxy-7,11,15(17)-cembratrien-16,14-olide [43], (3*E*,7*E*,11*E*)-18-acetoxy-3,7,11,15(17)-cembratetraen-16,14-olide [45], durumhemiketalolides A and C [46] from *Lobophytum durum*, sinulariol A from *Lobophytum crassum* [47], sarcocrassolide *from Sarcophyton crassocaule* [48], thioflexibilolide A from *Sinularia flexibilis* [49], dihydrosinularin and (−)14-deoxycrassin from *Sinularia triangular* [50], sarcocrassocolide I from *Sarcophyton crassocaule* [51], 11-dehydrosinulariolide and 11-epi-sinulariolide acetate from *Sinularia discrepans* [52].

At the same time, 13R-hydroxylobolide [44], durumolides B and D [44], G-D, L [45], durumhemiketalolide B [46] from *Lobophytum durum* and grandilobatin D from *Sinularia grandilobata* [53], 17-dimethylaminolobohedleolide from *Lobophytum crassum* [47], *s*arcocrassocolide A-E, sinularolide, 13-acetoxysarcocrassolide from *Sarcophyton crassocaule* [48], sinularin from *Sinularia triangular* [50], sarcocrassocolides F-H, J-L from *Sarcophyton crassocaule* [51], sarcophytolins A-B, D, but not C from *Lobophytum sarcophytoides* [54], crassarine H from *Sinularia crassa* [55] and sarcocrassocolides M-O from *Sarcophyton crassocaule* [56], querciformolide E from *Sinularia querciformis*, granosolide D, flexibilisolide A, flexilarin, sinulariolide, but not granosolide C and sinulaflexiolide E from *Sinularia granosa*, inhibited only iNOS protein expression [57], and crassarine F from *Sinularia crassa* inhibited only COX-2 protein expression [55]. Interestingly, no anti-inflammatory activity was found in macrophages under treatment with most of crassarines (B-E, G, H) from *Sinularia crassa* [55] and culobophylin A-C from *Lobophytum crassum* [58].

Anti-inflammatory activity was also abundant among eunicellin-based diterpenoids: simplexin E [59] and klysimplexin S [60], klysimplexin sulfoxide C [61] from *Klyxum simplex*, lymollin C-D, F-H [62] from *Klyxum molle*, which were active in reducing both iNOS and COX-2 protein accumulation, while simplexins A and D [59], klysimplexin J-N, R [60], klysimplexin sulfoxide A and B [61] from *Klyxum simplex*, hirsutalins C, D and H from *Cladiella hirsute* [63], lymollin B and E from *Klyxum molle* [62], krempfielins A-D, litophynol B and (1R*,2R*,3R*,6S*,7S*,9R*,10R*,14R*)3- butanoyloxycladiell-11(17)-en-6,7-diol [64] from *Cladiella krempfi* inhibited only NO generation.

For klysimplexins O-R [60], U-X from *Klyxum simplex* [65], krempfielin A from *Cladiella krempfi* [64], and hirsutalin E-G from *Cladiella hirsuta* [63], the iNOS and COX-2 inhibiting activity was not detected. It was shown that of eight verticillane-based diterpenoid cespitularins from *Cespitularia hypotentaculata* Cespitularin S possessed both iNOS and COX-2 expression inhibiting abilities, and cesputularins K, I, and F had only iNOS expression inhibiting activity [66]. Only one report examined norditerpenoids [66]. Of 18 compounds isolated from *Sinularia gyrosa,* gyrosanolides B and F, gyrosanin A, (1S*,5R*,8S*,10R*,11S*)-11-hydroxyl-1- isopropenyl-8-methyl-3,6-dioxo-5,8- epoxycyclotetradec-12-ene-10,12-carbonlactone, (1S*,5S*,8S*,10R*,11S*)-11-hydroxyl-1- isopropenyl-8-methyl-3,6-dioxo-5,8- epoxycyclotetradec-12-ene-10,12-carbonlactone, leptocladolide B and scabrolide D were able to inhibit iNOS protein accumulation. In addition, diterpenoids gyrosanols A and B were found to inhibit COX-2 protein expression [67].

Almost all numerous sterols studied inhibited iNOS and COX-2 protein accumulation: stoloniferone T and (25*S*)-24-methylenecholestane- 3β,5α,6β-triol-26-acetate from *Clavularia viridis* [68], chabrosterol from *Nephthea chabroli* [41], nebrosteroids D and F, G from *Nephthea chabroli* [69], griffinisterones F and G and griffinipregnone from *Dendronephthya griffini* [70], 1α,3β-dihydroxy-24*S*-methylcholesta-5-ene and 1α,3β-dihydroxy-24-methylenecholesta-5-ene from *Sinularia sp*. [71], 5,24(28)-ergostadien-3β,23*S*-diol and (22*S*)-5,24(28)-ergostadien- 3β,17α,22-triol from *Nephthea erecta* [72], nebrosteroids I-M from *Nephthea chabroli* [73], minabeolide-1,2,4,5 from *Paraminabea acronocephala* [74], and 8αH-3β,11-dihydroxy-5α,6α- expoxy-24-methylene-9,11- secocholestan-9-one from *Sinularia granosa* [75].

Stoloniferone S from *Clavularia viridis* [68], griffinisterones A-D from *Nephthea griffini* [76], nebrosteroids A-C, I from *Nephthea chabroli* [73], griffinisterone H *from Dendronephthya griffini* [70], 5,24(28)-ergostadien-3β,23*R*-diol and ergostanoid from *Nephthea erecta* [72], 5α,8α-epidioxy-22,23-methylene-24-methylcholest-6-en-3β-ol from *Lobophytum sarcophytoides* [54], paraminabeolides A-D from *Paraminabea acronocephala* [74], crassarosterosides A-C from *Sinularia crassa* [74], and 3β,11-dihydroxy-5β,6β-expoxy-24-methylene-9,11-secocholestan-9-one from *Sinularia granosa* [75] inhibited only iNOS expression. None of hirsutosterols A-G from *Cladiella hirsuta* [77] showed any anti-inflammatory activity.

Among the ceramides and cerebrosides, despite a lower number of them being studied, some were also found that had iNOS- and COX-2 inhibiting properties (ceramide from *Sarcophyton ehrenbergi*) or iNOS protein expression inhibiting activity (sarcoehrenoside A and cerebrosides 3-6 from *Sarcophyton ehrenbergi* [78]. Among other metabolites, capilloquinone and capillobenzopyranol, 2-[(2*E*,6*E*)-3,7-dimethyl-8-(4-methylfuran-2-yl)octa-2,6-dienyl]-5-methylcyclohexa-2,5-diene-1,4-dione, 2-[(2*E*,6*E*)-3,7-dimethyl-8-(4-methylfuran-2-yl)octa-2,6-dienyl]-5-methylbenzene-1,4-diol from *Sinularia capillosa* [79] and dihydroaustrasulfone alcohol from *Cladiella australis* [80] were found to inhibit COX-2 and/or iNOS expression.

Of note, all examined compounds studied were used at concentration of 10 μM, except for few reports. Thus, 20 and 50 μM concentrations of compounds were used in studies of Chen et al. [60] and Lu et al. [57]; additionally, concentration-dependent effects of compounds were evaluated in the work of Tseng et al. [39] at a concentration range of 1, 10, and 100 μM, and in the studies of Chen et al. [63] at concentrations of 2.5, 10, and 20 μM. For iNOS protein expression inhibiting capacity of cespitularins S from the Formosan soft coral *Cespitularia hypotentaculata*, the effective concentration 50(EC50s) was 7.80 ± 0.7 μM [66]. Once examined, no toxic effects of griffinisterones A-E to RAW264.7 cells were found at the 10 μM concentration, which is effective for inhibiting the upregulation of inflammatory and macrophage classical way markers [76].

In many laboratories, for inducing inflammation, LPS was used at concentration of 0.01 μg/mL, and time of incubation was 16 h [37,38,40,41,42,43,44,45,46,47,48,49,50,51,52,53,54,55,56,57,59,60,61,62,63,64,65,66,67,68,69,70,71,72,73,74,75,76,78,79]. In few cases, higher concentrations of LPS were used: 1 [39] or 10 μg/mL [36].

This indicates that, in most studies, there were no standard conditions of macrophage stimulation (a large enough concentration of LPS, known other activating stimuli and long-time incubation) and, in addition, there was lack of data on the phenotypes of macrophages during the experiment. Nevertheless, these studies were useful for generating basic libraries of anti-inflammatory activities from a broad spectrum of soft corals for further research. In case of using standard conditions (LPS concentration, known other activating stimuli and incubation time) for macrophage activation, more reasoned conclusions may be made. Thus, although briarane-based diterpenoids were not shown to be studied for the iNOS and COX-2 inhibiting activities in the review of Wei et al. [33], briarane-type diterpene xcavatolide B (BrD1, 20 μM), isolated from a Formosan gorgonian coral *Briareum excavatum* (Nutting, 1911), was shown to inhibit LPS (100 ng/mL)-induced expression of pro-inflammatory cytokines IL-6 and, at lesser extent, TNF-α, in mouse bone marrow-derived dendritic cells after 24 h [81].

**Figure 1:**
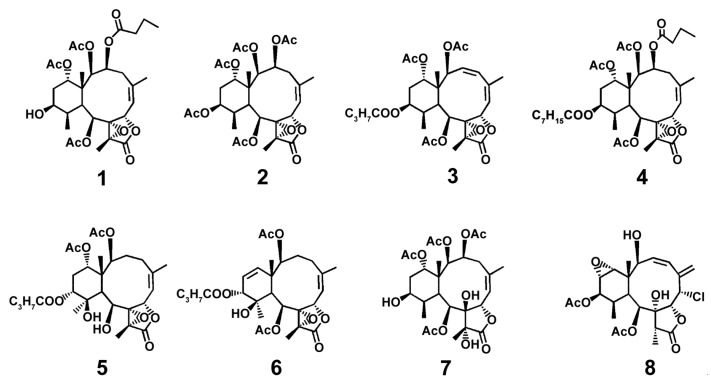
Chemical structure of the briarane-based diterpenoids. (**1**) excavatolide B (BrD1); (**2**) excavatolide K (BrD2); (**3**) excavatolide F (BrD3); (**4**) briaexcavatolide R (BrD4); (**5**) excavatolide Z (BrD5); (**6**) briaexcavatolide B (BrD6); (**7**) briaexcavatolide K (BrD7); (**8**) briaexcavatolide H (BrD8).

Authors explain such differences between TNF-α and IL-6 inhibition by more early and rapid TNF-α expression resulted from LPS-induced TNF-α mRNA splicing [82]. Additionally, comparison of cytokine inhibiting properties of different briarane-based diterpenoids (Figure 1) revealed that a presence of 8, 17-epoxides of BrDs is crucial for the effects of compounds on LPS-induced expression of the cytokines, but the presence of an α position of the functional group at C-12 and the steric hindrance of longer acyloxyl groups impaired the capacity of compounds to inhibit LPS-induced cytokine expression. Analysis of the conditions of the experiment indicates that LPS exposure resulted in increase of M1-macrophage marker expression, and BrD1 can be considered potential to shift the M1-phenotype towards the M2-type.

Later, excavatolide B at concentrations of 1, 10, 25, and 50 μM was shown to inhibit iNOS protein expression, and at concentration of 50 μM inhibited COX-2 expression compared to those induced by LPS alone in RAW 264.7 cells [83]. These data confirm the suggestion about the promising anti-inflammatory properties of the compound, with a mechanism involving the influence on the macrophage phenotype.

**Figure 2:**
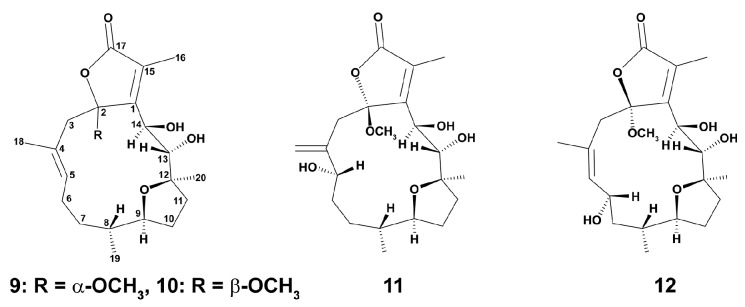
Structures of briaviodiol F (**9**), briaviotriols A (**10**) and B (**11**), and briaviodiol A (**12**).

More recent studies on the cembrane-type diterpenoids of another Taiwan species of the same family Briareidae, *Briareum violaceum* (Quoy and Gaimard, 1883) were carried out in RAW 264.7 cells stimulated for 16 h with LPS at a concentration of 10 μM [84]. Only cembranoids **10**–**12** (10 μM) (Figure 2) exerted inhibition effects on iNOS release from the cells, and briaviotriol A (**11**) and briaviodiol A (**10**) were shown to be the most potent suppressors of iNOS release, suggesting their potential in shifting the M1 phenotype towards the M2 type of macrophages.

**Figure 3:**
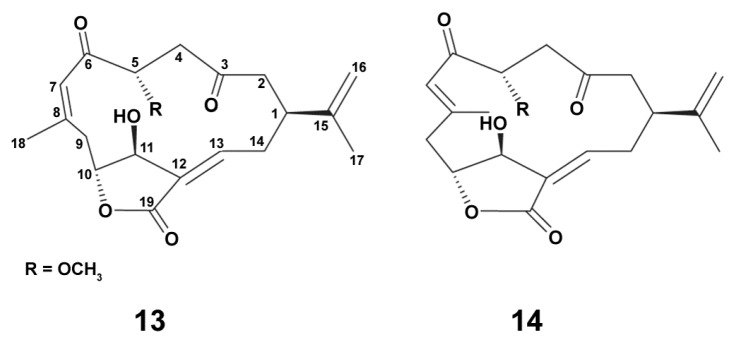
Structure of sinumerolide A (**13**) and its epimer, 7E-sinumerolide A (**14**).

Two new cembrane diterpenoid norcembranoids, sinumerolide A (**13**) and its epimer, 7*E*-sinumerolide A (**14**), were isolated from the ethyl acetate extract of the Taiwan soft coral *Sinularia numerosa* (Tixier-Durivault, 1970). In LPS (10 ng/mL)-induced for 16 h RAW264.7 macrophage cells, compounds (Figure 3) **13** and **14** (10 μM each) significantly inhibited LPS-induced iNOS, but not COX-2 protein expression in macrophages [85]. Dexamethasone (10 μM) was used as a positive control as it is known as an anti-inflammatory agent, and is additionally able to modulate macrophage polarization via M2-way [44]. In this experiment, dexamethasone inhibited both iNOS and COX-2 protein expression compared to the effects of LPS alone. These data apparently indicate that the both compounds are able to exert their anti-inflammatory effects via influence on macrophage polarization, but with the mechanisms which are distinctly different from those of dexamethasone.

**Figure 4:**
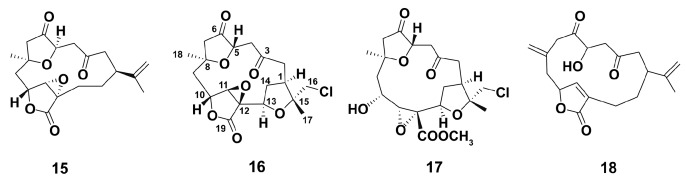
Chemical structure of macrocyclicditerpenoids scabrolide D (**15**), chloroscabrolides A (**16**) and B (**17**), and prescabrolide (**18**).

Chlorinated compounds are rare among soft coral metabolites [86]. Studies on antiinflammatory activities of chlorinated norcembranoids (Figure 4) from the Indonesian soft coral *Sinularia* sp. [86] were conducted in J774 cells stimulated with LPS (1 mg/mL) for 24 h. Almost all compounds were inactive in relation to inhibition of LPS-stimulated NO production, and only scabrolide D slightly decreased this marker expression. Obviously, chlorinated norcembranoids are not promising agents to regulate macrophage balance, presumably due to a chlorine atom.

**Figure 5:**
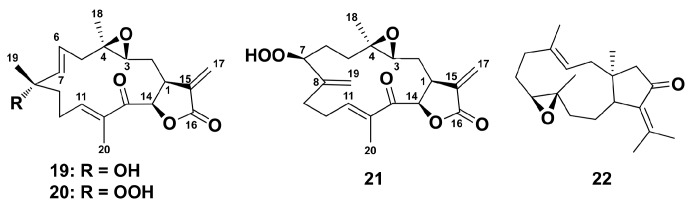
Structure of diterpene uprolide N (**19**), uprolide O (**20**), uprolide P (**21**), and dolabellane (**22**).

Octocorals from the genus *Eunicea* have to be a prolific source of chemically complex diterpenoids, including recently isolated group of highly functionalized α-methylene-γ-lactone cembranolides, characterized by the presence of a Δ6 olefin known as the uprolides [87]. In LPS (100 μg/mL, 6 h incubation)-stimulated primary murine macrophages from C57B1/6 mice, compounds **19**–**21**, but not **22**, (Figure 5) inhibited the production of TNFα and IL-6 in a dose-dependent manner, the compounds were unable to induce the production of TNF and IL-6 in the absence of LPS. Apparently, uprolides **19**–**21** may be considered promising to stimulate macrophage polarization via M2-type way.

**Figure 6:**
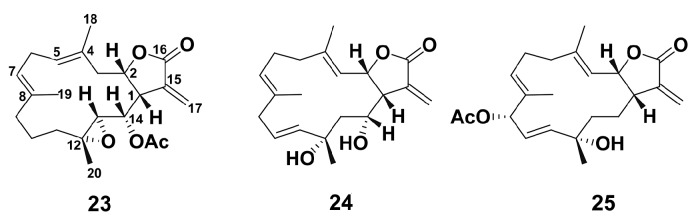
Structure of new cembranolides (**23**)–(**25**) from an Okinawan soft coral, *Lobophytum* sp.

In addition, three new cembranolides (**23**–**25**) (Figure 6) which were isolated from an Okinawan soft coral, *Lobophytum* sp., were evaluated for anti-inflammatory effect in LPS-stimulated inflammatory RAW 264.7 macrophage cells [88]. All compounds, especially compound **23**, significantly decreased NO production in LPS-stimulated cells.

**Figure 7:**
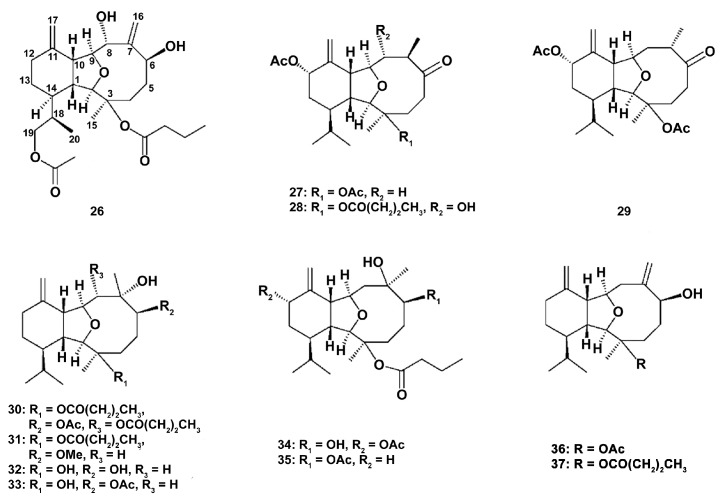
Structure of eunicellin-based diterpenoids krempfielins E–I (**26**)–(**30**), 6-methyl ether of litophynol B (**31**), sclerophytin A (**32**), sclerophytin B (**33**), litophynin I monoacetate (**34**), 6-acetoxy litophynin E (**35**), (1R*,2R*,3R*,6S*,9R*,10R*,14R*)-3-acetoxycladiell-7(16),11(17)-dien-6-ol (**36**), and litophynin F (**37**).

Studies on five new eunicellin-based diterpenoids krempfielins E–I (**26**–**30**) along with the known compounds 6-methyl ether of litophynol B (**31**), sclerophytin A (**32**), sclerophytin B (**33**), litophynin I monoacetate (**34**), 6-acetoxy litophynin E (**35**), (1R*,2R*,3R*,6S*,9R*,10R*, 14R*)-3-acetoxycladiell-7(16),11(17)-dien-6-ol (**36**), and litophynin F (**37**) (Figure 7) from the Taiwanese soft coral *Cladiella krempfi* (Hickson, 1919) have shown that new compounds were not active, and only 6-methyl ether of litophynol B (**31**) could inhibit both the accumulation of the pro-inflammatory iNOS and the expression of COX-2 protein in LPS-stimulated RAW264.7 macrophages [89]. 6-acetoxy litophynin E (**35**) was able to inhibit iNOS, and litophynin F (**37**) inhibited the COX-2 protein expression. These findings demonstrate that the compounds influence the different stages of the cell response, and, apparently, compounds **31** and **35** may be used as potent inhibitors of classical macrophage activation.

Immunomodulating activity of polysaccharides of soft corals is lesser studied compared to other metabolites [90]. Polysaccharides isolated from *Pseudopterogorgia americana* (Gmelin, 1791) [90] induced the expression of TNF-α, IL-6, and COX-2 in mouse macrophages, but had no effect on the expression of iNOS protein and NO production. However, polysaccharides reduced expression of TNF-α and IL-6 in LPS-activated macrophages through the downregulation of interleukin-1 receptor-associated kinase (IRAK)2 expression, mitogen-activated protein kinase (MAPK) phosphorylation, and NF-κB activation. Apparently, polysaccharides alone do not activate polarization macrophages in concentrations studied, as the main marker of M1 macrophages, NO level, was not changed; however, they decrease expression of proinflammatory cytokines in LPS-activated macrophages that may indicate that these compounds may be potent in reprograming the polarization of macrophages via the M1-type way.

#### 2.1.2. Sea Anemones

**Figure 8:**
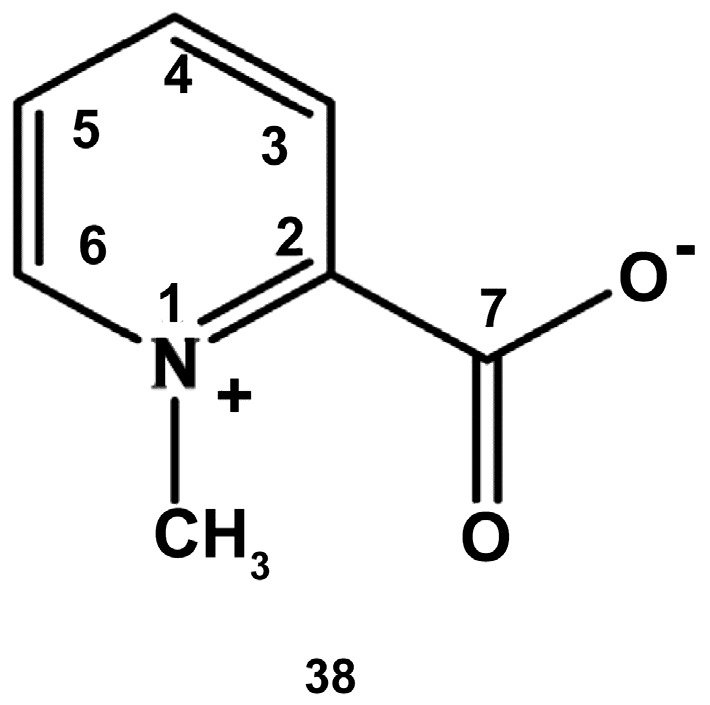
Structure of homarine.

Sea anemones (Actiniaria) is an order of cnidarians belonging to class Anthozoa, subclass Hexacorallia. They remain poorly studied regarding their potential anti-inflammatory effects [91]. The presumable activities of water extracts from two species, *Actinia equina* (Linnaeus, 1767) and *Anemonia sulcata* (Pennant, 1777) from Portugal, were examined in RAW264.7 cells. Both preparations were able to decrease the NO levels in LPS (1 μg/mL)-treated cells (22 h of incubation), but at different concentrations. *A. sulcata* inhibited it in a dose-dependent way with the half maximal inhibitory concentration (IC_50_) of 0.374 mg/mL, and *A. equina*—at concentrations of 0.125 mg/mL, 0.25 and 0.5 mg/mL (IC_50_ was not determined). The purine alkaloid homarine (**38**) (Figure 8), the major metabolite found in both extracts, also reduced NO levels. The enhanced level of ROS, another marker of macrophage activity, was decreased by both extracts, but not homarine, at a 0.0625 mg/mL concentration. Both extracts and homarine also significantly inhibited activity of PLA2, which is responsible for production of prostaglandin E2 [6], the extract from *A. equina* and homarine (each at concentrations of 0.5 and 1 mg/mL) being more potent compared to the extract of *A. sulcata.* These facts indicate that the extracts and homarine may inhibit an early stage of the signaling pathway leading to M1-type macrophages. In relation to such parameters studied, as NO production and PLA2 inhibition, homarine displayed significant activity that apparently could be responsible for the macrophage polarization, but not antioxidant activity of the extracts. Hence, the extracts and homarine have a potential in modulating macrophage polarization and redirecting it towards the M2 phenotype.

### 2.2. Porifera

The phylum Porifera contains about 5500 species, which are considered the most prolific regarding their pharmacological potential. They are a rich source of a variety of secondary metabolites including terpenoids, macrolides, and sterols [92]. Nevertheless, studies on their anti-inflammatory activities are mostly at the early stage.

Methanol extracts of *Hyrtios* and *Haliclona* species (Demospongiae), collected near Kosrae Island, Micronesia, Central Pacific, were examined for their activities in the LPS (1 μg/mL)-treated for 24 h Raw 264.7 cells [93]. Both extracts (25, 50, and 100 μg/mL each) exhibited dose-dependent ability to reduce NO generation, and at concentration of 10 μg/mL, the extracts inhibited the proinflammatory cytokine IL-1β production, whose level characterizes M1 macrophage phenotype [12]. This research shows the potential of the extracts as anti-inflammatory drug candidates with macrophage polarizing properties.

Similarly, ethanol extracts from the species of two other sponge genera, *Mycale* (*Oxymycale*) *acerata* (Kirkpatrick, 1907), *Isodictya erinacea* (Topsent, 1916), and *Isodictya toxophila* (Burton, 1932) collected from the Eastern Weddell Sea and the South Shetland Islands were used for screening their impact on inflammatory mediators at non-toxic concentrations (50, 125, and 250 μg/mL for each species) in the LPS (1 μg/mL)-stimulated Raw 264.7 cells [94]. The effects of the extracts were distinct regarding different markers. Thus, the extract of *Isodictya erinacea* inhibited significantly and dose-dependently the release of IL-1β and PGE_2_, although not affecting LTB_4._ The extracts of the sponge *Isodictya toxophila* decreased the release of IL-1β and LTB_4_ at two non-cytotoxic concentrations (50 and 125 μg/mL) and also diminished PGE_2_ release at the intermediate concentration (125 μg/mL). *Mycale* (*Oxymycale*) *acerata*’s extract decreased the release of all inflammatory mediators studied IL-1β, PGE_2_, and LTB_4_ at the lower concentration of 50 μg/mL. Taking into consideration, the role of these mediators in the mechanisms of polarization to M1-type macrophages [12], the extracts, especially that from *Mycale* (*Oxymycale*) *aceratas*, seem to have potential for pharmaceutical application as macrophage targeting agents.

The extract [dichloromethane/methanol (1:1)] (100 µg/mL) of another species of the *Isodictya* genus, *Isodictya palmata* (Ellis & Solander, 1786), inhabiting the North Atlantic, was also shown to reduce the release of pro-inflammatory cytokines (IL-12 and IL-10) in human monocyte-derived dendritic cells [95]. Moreover, this study revealed that the extract reduced CD86 expression. CD86 is one of the cell surface markers for M1-type macrophages [96], and the findings clearly demonstrate the involvement of polarization of macrophages to an M2 type in the mechanisms of the influence of the extract on macrophages and indicate that the extract has potential in application as a macrophage-targeted anti-inflammatory drug.

**Figure 9:**
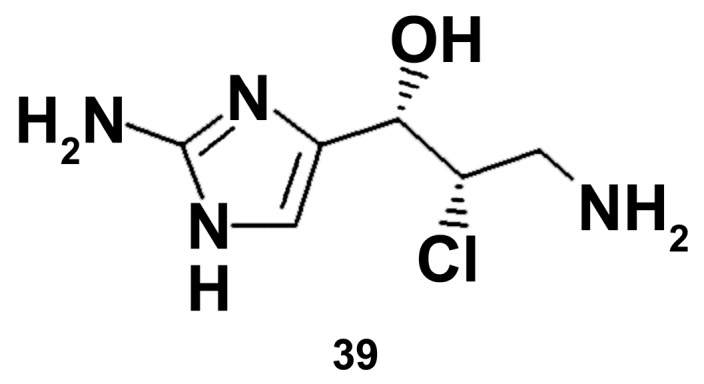
Structure of girolline.

The identified compound girolline (**39**) (Figure 9), isolated from the marine sponge *Pseudoaxinyssa cantharella* (Demospongiae), was evaluated for its anti-inflammatory activity using the model of stimulation of cell response by flagelline-induced activation of the Toll-like receptor (TLR), followed by the activation of the nuclear transcription factor NF-κB and the production of proinflammatory cytokines [96]. Girolline (2 μg/mL) almost completely abolished NF-κB activity in THP1-derived macrophages and decreased the secretion of the pro-inflammatory cytokine IL-6 in both flagellin-treated human peripheral blood mononuclear cells and THP1-derived macrophages [97]. Elongation factor 2 was defined to be a molecular target of girolline. It is noteworthy that flagelline is known to be the TLR5 ligand, while LPS is TLR4 agonist [98]. Apparently, the data obtained with girolline on this model need to be proved with a well-known inducer of macrophage polarization to be sure on its potency as macrophage polarization modulator.

### 2.3. Mollusca

**Figure 10:**
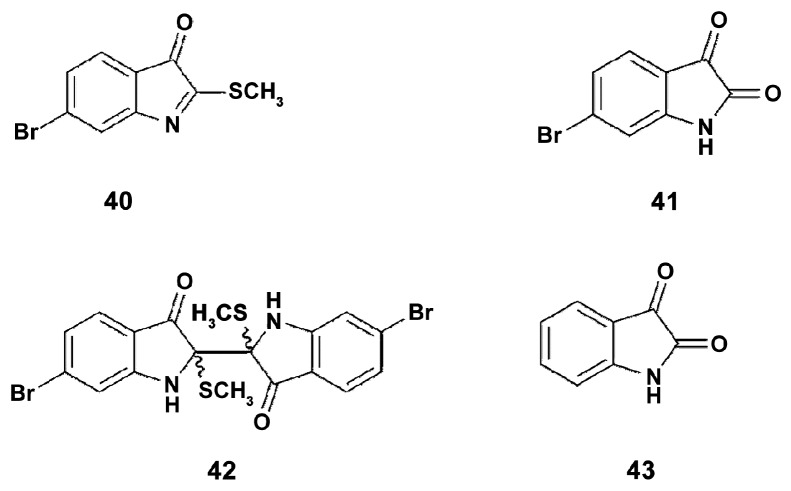
The structure of tyrindoleninone (**40**), 6-bromoistain (**41**), tyriverdin (**42**), and isatin (**43**).

Phylum Mollusca contains about 50,000 species [99], and only a small proportion of them were used as a source for anti-inflammatory preparations and/or were tested for in order to highlight their immunomodulatory properties [100]. The muricidae molluscs are known for their production of the Tyrian purple dye, and some of the dye precursors, e.g., tyrindoleninone (Figure 10), with specific anticancer activity [3]. Hypobranchial gland and egg chloroform extracts, and brominated indoles from the Australian muricid *Dicathais orbita* (Gmelin, 1791) (Gastropoda, Neogastropoda), were tested on LPS-stimulated RAW264.7 macrophages for the capacity to modulate expression of the markers of inflammation. Both hypobranchial gland and egg chloroform extracts inhibited the production of NO in LPS-stimulated RAW264.7 macrophages with IC_50_ of 30.8 μg/mL and 40 μg/mL, respectively. The hypobranchial gland extract also inhibited the production of TNFα with IC_50_ of 43.03 μg/mL. Isolated from the extract tyrindoleninone (**40**) and tyriverdin (**42**) were tested along with a range of synthetic brominated indole derivatives, and mono-brominated indole (**41**) was shown most active in inhibition of NO production, which indicates that the position of the bromine atom on the isatin (**43**) benzene ring is important for immunomodulating activity. The chemical structure of compounds is shown in Figure 10. Apparently, brominated indoles from muricids are potent for use in macrophage reprogramming.

*Aplysia depilans* (Gmelin, 1791) (Gastropoda, Aplysiida) is distributed in the Mediterranean Sea and in the Atlantic Ocean. In the methanol extract from *A. depilans* twenty-two fatty acids, *cis*-5,8,11,14-eicosatetraenoic (arachidonic acid), being the major one, and eight carotenoids (zeaxanthin as the major xanthophyll, fucoxanthin and two isomers, neoxanthin, lutein, zeaxanthin, and α- and β-carotene) were identified [101]. The extract of the *A. depilans* digestive gland was able to decrease the NO level in a dose-dependent way (IC_50_ value of 0.663 mg/mL) in LPS (1 µg/mL) -exposed RAW 264.7 cells, incubated for 18 h. This suggests that the anti-inflammatory capacity of the extract may be due to the inhibition of iNOS expression and, possibly, involves inhibition of macrophage polarization through the M1-way. The anti-inflammatory activity of the *A. depilans* digestive gland obviously may be partially resulted from the activity of the metabolites identified, mainly with unsaturated fatty acids and carotenoids. *Cis*-5,8,11,14-eicosatetraenoic, being n-6 polyunsaturated fatty acid (PUFA), is considered a pro-inflammatory fatty acid, once it is a precursor of the synthesis of eicosanoids, increasing the inflammatory response [102]. On the other hand, long-chain n-3 PUFAs, such as *cis*-5,8,11,14,17-eicosapentaenoic (EPA) and *cis*-4,7,10,13,16,19-docosahexaenoic (DHA) acids, are known also to inhibit iNOS expression [103], decrease the production of inflammatory mediators (eicosanoids, cytokines and NO) [102], and shift M1/M2 macrophage balance towards the M2 phenotype [32]. Carotenoids identified in this work were shown earlier to influence the expression of the markers of inflammation and macrophage polarization. Thus, zeaxanthin and fucoxanthin inhibited the NO production, as well as expression of iNOS, COX-2, and pro-inflammatory cytokines [104]. Hence, the extract may be considered a potent M1–M2 modulator due to its constituents’ fatty acids and carotenoids identified.

The anti-inflammatory preparation Lyprinol^®^ is an example of practical application of fatty acid-rich extract from mollusks. Lyprinol^®^ is prepared from New Zealand green-lipped mussel *Perna canaliculus* (Gmelin, 1791) (Bivalvia, Mytilida) and contains five main lipid classes including sterol esters, triglycerides, sterols, polar lipids and free fatty acids, mainly EPA and DHA, and also carotenoids. Lyprinol subfractions inhibit PGE2 production by activated macrophages [105]. Moreover, in human THP-1 monocytes activated by a combination of IFN-γ (10 ng/mL) and LPS (1 μg/mL) during 16 h incubation, the extract reduced the production of pro-inflammatory cytokins TNF-*α* and IL-12 [106]. As the standard combination for activation of M1 stage was used in this study, the ability of the extract to decrease the level of M1-macrophage markers demonstrates that the preparation can promote switching macrophage polarization towards M2-type macrophages.

The influence of the ratio of n-3 PUFA and n-6 PUFA for anti-inflammatory activity of the preparation was demonstrated in the studies on the Mediterranean molluscs belonging to different genera: *Armina maculata* (Rafinesque, 1814) and *Armina tigrina* (Rafinesque, 1814) (Gastropoda, Nudibranchia) on the one side and *Aglaja tricolorata* (Renier, 1807) (Gastropoda, Cephalaspidea) on the another [107]. In addition, 23, 26, and 18 fatty acids (FA) were identified in the extracts of *A. maculata, A. tigrina*, and *A. tricolorata*, respectively. *A. tricolorata*’s extract exhibited higher proportion of n-3 PUFA (n-6/n-3 < 1) in contrast with the *A. maculata* and *A. tigrina* with a n-6/n-3 ratio higher than 1.75. The significantly higher reduction of NO levels was shown in LPS-challenged RAW 264.7 macrophages exposed to *A. tricolorata*’s extract compared to the effects of the extracts of two other species. Since n-3 PUFA have been reported as anti-inflammatory agents influencing cell signaling and macrophage polarization [103,104], the higher proportion of those FA in *A. tricolorata*’s extract is a possible reason of such different effects of extracts studied.

A non-standard approach to evaluate the compound bioactivity was suggested in the case of bone remodeling when two opposite physiological processes are executed by the two types of cells. Osteoclasts and osteoblasts play opposite roles in this process and osteoclasts are considered a primary target for the treatment of bone resorption [108]. To test the capacity of fermented extract (FO) of the Pacific oyster *Crassostrea gigas* (Thunberg, 1793) (Bivalvia, Ostreida) to prevent osteoclast differentiation, induced by the receptor activator of NF-κB ligand (RANKL), the RAW 264.7 cells were used as osteoclast precursors. ROS are known to promote the transcription of osteoclast-specific genes in RANKL-induced osteoclasts [109]; therefore, the ROS level is an important marker of an osteoclast functional phenotype. FO was shown to decrease RANKL-stimulated ROS production and to inhibit RANKL-induced nuclear translocation of NF-κB in RAW 264.7 cells. Taking into consideration that FO inhibited the rise of markers which characterize the polarization of macrophages via M1-type (ROS, nuclear translocation of NF-κB), it can be suggested that FO is able also to prevent classical macrophage polarization induced through the NF-κB-associated ways. However, this suggestion must be verified in the standard conditions with using the known activators of macrophage polarization towards the M1-type.

Recently, the most fundamental studies of the anti-inflammatory mechanisms of mollusk-derived preparations were reported by Dong et al. [110]. The water extract of widely distributed in the world ocean scallop *Atrina pectinata* (Glenn, 1904) (Bivalvia, Ostreida) was tested for anti-inflammatory activity in LPS (250 ng/mL)-stimulated RAW 264.7 cells. The scallop extract effectively reduced the synthesis of NO, ROS generation and the expression of IL-6 and TNF-α. The mechanisms of such reduction were examined and highlighted as related to downregulation of MAPK (JNK, p38 and ERK) and NF-κB signaling. According to these findings, mechanisms of the anti-inflammatory effect of the extract were realized via the same ways that are described for polarization of macrophages towards the M2-type.

**Figure 11:**
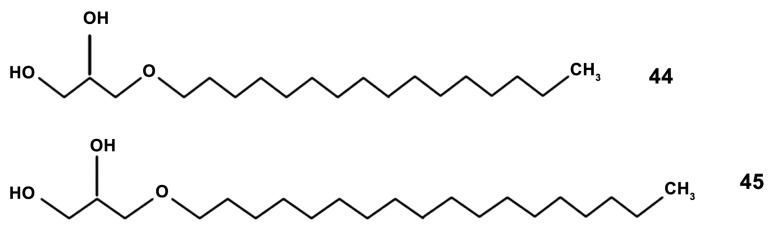
Structures of the alkyl glycerols: (**44**) chimyl alcohol; (**45**) batyl alcohol.

In addition to PUFA, the digestive glands of some mollusk species contain a large amount of ether lipids 1-*O*-alkyl-2,3-diacyl-sn-glycerols which are formed by fatty acids and fatty alcohols (compounds **44**, **45** in Figure 11). The 1-*O*-alkyl-sn-glycerols were isolated from the digestive gland of the squid *Berryteuthis magister* (Berry, 1913) (Cephalopoda, Oegopsida). This compound was described to cause an increase in the level of ROS and the synthesis of NO and IL-6 expression in the RAW264.7 cell line at non-toxic concentrations of 0.1–5 µg/mL [111]. This effect was comparable with that produced by LPS (1 µg/mL). This implies that the compound may be a promising immunostimulant agent. However, further research has to be performed for understanding whether macrophage polarization mechanisms are involved in this effect.

### 2.4. Crustacea

The sub-phylum Crustacea (Arthropoda) holds a massive biodiversity as it includes about 68,000 species [99]. Lipids of crustaceans are mostly studied for their bioactive properties [112]. However, classes of Crustacea are distinctly different from each other regarding the main constitutive compounds. Thus, more than 80% of marine copepod *Calanus* oil consists of wax esters, i.e., long-chain fatty alcohols linked to long-chain fatty acids [113]. Lipids of shrimps consist of free fatty acids, triglycerides, carotenoids and other lipids [114]. Carotenoids include astaxhantin (3,3’-dihydroxy-β, β’-carotene-4,4’-dione), astaxanthin esters, β-criptoxanthin, α-carotene, β-carotene, meso-zeaxanthin, canthaxanthin, lutein, zeaxanthin and crustacyanin, and some of these compounds were reported to have anti-inflammatory and macrophage activity modulating properties [115]. Crabs (Decapoda) are essentially constituted by xanthophylls, astaxanthin esters being the main compounds [116].

Thus, lipids of the Antarctic krill *Euphausia superba* (Malacostraca) are rich in n-3 PUFA EPA (12%) and DHA (7%) [117]. The high content of the two biologically active components, EPA and DHA, are responsible for the majority of physiological effects of krill oil [118].

The anti-inflammatory mechanisms of FlexPro MD^®^ (FP-MD), a novel multi-ingredient dietary supplement, consisting of a mixture of krill oil, astaxanthin and hyaluronic acid were studied in RAW264.7 macrophage cells challenged with l μg/mL LPS [119]. FP-MD significantly inhibited the mRNA levels of pro-inflammatory cytokines IL-6, TNF-α and IL-1β, and, in contrast, it elevated the mRNA levels of anti-inflammatory cytokine IL-10. In addition, FP-MD reduced LPS-induced phosphorylation levels of NF-κB p65 and inhibitor of κB-α (IκB-α). The changes in the functional markers indicate that FP-MD could shift the macrophage balance by increasing M2 type activity and inhibiting the M1-type in RAW264.7 macrophage cells, and, most possibly, carotenoids and n-3 PUFA contributed to this effect. The expression levels of pro-inflammatory cytokines and inflammatory markers also reduced in mice with LPS-induced inflammatory arthritis after treatment with FP-MD that suggests possibility of involvement of the mechanisms including inhibiting NF-κB in the anti-inflammatory effects of FP-MD.

Similarly, krill oil from *Euphausia superba* (80 mg/kg/day for one month) [117] effectively inhibited the expression of iNOS and cyclooxygenase-2 COX-2 induced by the administration of LPS (250 μg/kg, 7 days) and decreased ROS and malondialdehyde levels in mice brain. Studies of the major components of krill oil EPA and DHA in microglia (CNS macrophage)-like BV-2 cell line revealed that these PUFAs dose-dependently decreased LPS (1 μg/mL)-induced nitric oxide and ROS generation, COX-2 and iNOS expression as well as NF-κB activity in model macrophages after 24 h of incubation. These results confirm the data on the macrophage-polarization modulating capacity of n-3 PUFA [102] and suggest that anti-inflammatory effect of krill oil in mice may result from its capacity to negatively influence classical activation of microglial cells. However, further studies in vivo are necessary to clarify the mechanisms of the anti-inflammatory effects of krill oil, as the direct inhibition of Th1 activity, possibly, through induction of apoptosis, but not increase in polarization of T-cells towards Th2 phenotype, associated with macrophage polarization, was reported at least ex vivo [120].

A focus on carotenoid ingredients of shrimps was made in the studies of anti-inflammatory properties of a shrimp waste extract [121]. Comparison of shrimp extract and commercial astaxanthin showed a suppressive effect of those preparations on the generation of O_2_(-), NO and TNF-α secretion in LPS-induced rat alveolar macrophages, while purified shrimp astaxanthin did not suppress O_2_(-) production, and the effect of shrimp extract on TNF-α secretion was more pronounced. These findings support the suggestion about the ability of the shrimp extracts to influence macrophage functional phenotypes through an alternative way, and indicate that it may be partly caused by the presence of astaxanthin.

Crustaceans of the order Decapoda (crab, shrimp, prawn, and lobster) are also a valuable source of chitin [122]. Being isolated from tissues, purified chitin is a plain polysaccharide. There is a large body of knowledge on the use of chitosans as biomaterials, and, apparently most important, as drug carriers. However, of special interest is the fact that some of the structural constituents of chitosan may modulate the effects of other molecules, which can basically promote the drug delivery targeting M1 or M2 macrophages dependent on the aim of application. Thus, treatment of RAW264.7 macrophages with a combination of chitosan hydrolysate/chitooligosaccharide mixture and IFN-γ significantly induced NO production in a dose-dependent manner, while a low-molecular weight chitosan inhibited NO production in combination with IFN-γ [123]. Both hydrolysate and chitooligosaccharide mixture through binding to the receptors of CD14, TLR4, which are the markers of M1 macrophages [8,96], promoted the migration of NF-κB into the nucleus, and a specific inhibitor of NF-κB blocked NO production. Apparently, different chitosan derivatives may modulate the effects of inflammation mediators in a different manner, may be, through distinct capacities in binding the cell surface receptors/changing the cell phenotype. Taking into consideration that tumor-associated macrophages (TAMs) have the protumoral functions, and their polarization via M2-type way is important for tumor progression [124], the capacity of the compounds to induce proinflammatory way of macrophage activation indicates a potential of these compounds in treatment of cancer.

### 2.5. Echinodermata

Some of the most familiar seashore animals are members of the phylum Echinodermata. The phylum contains about 7000 living species, including the crinoids, asteroids (sea stars), ophiuroids (brittle stars), echinoids (sea urchins), and holothurians (sea cucumbers) [99].

#### 2.5.1. Crinoidea

**Figure 12:**
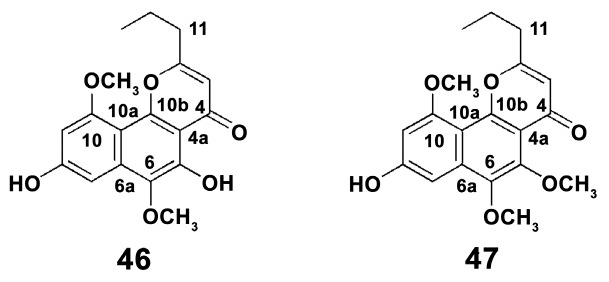
Structure of naphthopyrones of the *Comanthus parvicirrus*. (**46**) 6-methoxycomaparvin; (**47**) 6-methoxycomaparvin 5-methyl ether.

To date, there are about 600 species of crinoids [125]. Their small number and mostly deep-sea habitat apparently result in a lack of studies on their anti-inflammatory capacities. Attention was mostly paid to naphthopyrone compounds. The two naphthopyrones 6-methoxycomaparvin (**46**) and 6-methoxycomaparvin 5-methyl ether (**47**) were isolated from a bioactive methanol-soluble extract of the Fijian crinoid *Comanthus parvicirrus* (Müller, 1841) (Figure 12) [126]. It was shown that these compounds completely inhibit TNF-alpha-induced NF-κB activation in the leukemia cell line K562 at a relatively high concentration of 300 μM. This indicates possible slight anti-inflammatory effect of those substances.

**Figure 13:**
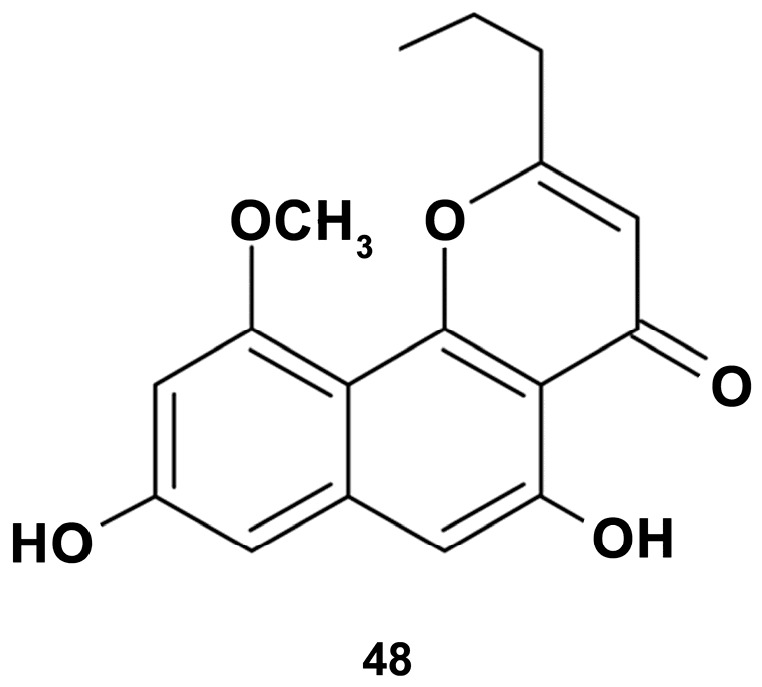
Structure of comaparvin.

Further studies on the bioactivity of the species of the same genus *Comanthus bennetti* revealed that the crude extract significantly inhibited the expression of pro-inflammatory proteins in LPS-stimulated RAW 264.7 cells [127]. Isolated from the extract comaparvin (5,8-dihydroxy-10-methoxy-2-propylbenzo[h]chromen-4-one) (compound **48** in Figure 13) at concentrations of 1, 10, 25, and 50 μM dose-dependently decreased the expression of iNOS protein and mRNA in LPS (0.01 μg/mL)-stimulated for 16 h macrophage cells, but did not inhibit cyclooxygenase-2 (COX-2) expression. Apparently, the comaparvin-induced anti-inflammatory effects observed show downstream COX-2 expression. However, changes in the marker of M1 macrophage suggest that the mechanisms of the anti-inflammatory effect of the compound may involve the inhibition of classical activation of macrophages.

#### 2.5.2. Asteroidea

Sea stars contribute to Echinoderm variety with about 2000 species [128]. A lot of its structurally variable secondary metabolites attract the attention of researchers; however, little is known about their immunomodulatory capacities.

Studies on the lipidomic profiling of the sea star *Marthasterias glacialis* (Linnaeus, 1758), collected in west Portugal, revealed two major classes of compounds: fatty acids and sterols [129]. Among those, the predominant compounds were cis-11-eicosenoic and cis-11,14 eicosadienoic acids, and, additionally, the unsaturated sterol ergosta-7,22-dien-3-olas. These compounds as well as lipophilic extract were evaluated for their anti-inflammatory effects in the LPS (1 μg/mL)-induced RAW 264.7 macrophages model of inflammation for 24 h. In these studies, the mechanisms involved in the immunomodulating effects of metabolites were studied in more detail compared to those of any other marine compounds reported above. The extract significantly diminished the levels of LPS-induced NO as a result of downregulation of iNOS, an effect for which all compounds tested contributed, notably ergosta-7,22-dien-3-ol. However, only the extract, but not single compounds, prevented an LPS-induced increase of the expression of COX-2, IL-6 level, and ROS generation. The extract and some of its constituents also inhibited the activation of the NF-kB pathway, which is the trigger of the inflammatory response, with subsequent ROS and ER-stress marker elevation [3]. In this study, ER-stress status was also evaluated by the expression of the endpoint protein of the CHOP, an ER-stress marker [5]. It is noteworthy that CHOP damages the RAW 264.7 cells largely dependent on pro-inflammatory stimuli used [130]. In this study, LPS increased CHOP expression levels, and this effect was partly reverted by the extract and its components, indicating the compounds could attenuate NF-kB activation and subsequent expression of some inflammatory markers. Apparently, the mechanisms of action of the compounds are distinct as they modulated different levels of the inflammation pathway. However, the fact that all of them were active in NO level inhibition indicates that the extract could inhibit the classical activation of macrophages, and its constituents contribute to this effect.

Steroidal glycosides are the predominant metabolites of starfish, and are responsible for their general toxicity [131]. Steroidal glycosides, protolinckiosides A-D (1-4, respectively) were isolated from the MeOH/EtOH extract of the tropical starfish *Protoreaster lincki* (Blainville, 1834). The structures of 1-4 were elucidated as (3β,4β,5α,6β,7α,15α,16β,25S)-4,6,7,8,15,16,26-heptahydroxycholestan-3-yl 2-*O*-methyl-β-d-xylopyranoside (1), (3β,5α,6β,15α,24S)-3,5,6,8,15-pentahydroxycholestan-24-yl α-l-arabinofuranoside, sodium (3β,6β,15α,16β,24R)-29-(β-d-galactofuranosyloxy)-6,8,16-trihydroxy-3-[(2-*O*-methyl-β-d-xylopyranosyl)oxy]stigmast-4-en-15-yl sulfate and sodium (3β,6β,15α,16β,22E,24R)-28-(β-d-galactofuranosyloxy)-6,8,16-trihydroxy-3-[(2-*O*-methyl-β-d-xylopyranosyl)oxy] ergosta-4,22-dien-15-yl sulfate. All compounds significantly decreased the ROS content in LPS-challenged RAW 264.7 macrophages. These results are too limited to discuss the participation of the compounds in macrophage –polarization mechanisms.

**Figure 14:**
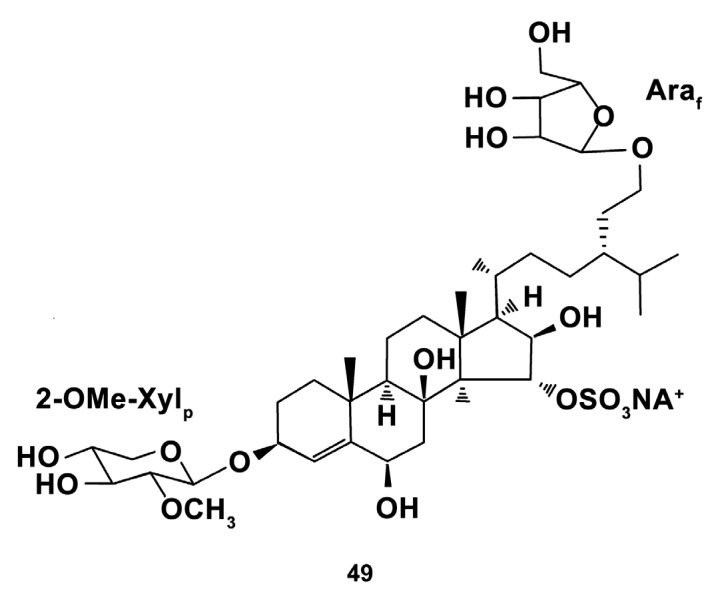
Structure of the granulatoside D.

In addition, unique steroid glycosides, polyhydroxysteroids capelloside A, and oscinasteroside (24S)-24-*O*-(3-*O*-methyl-β-d-xylopyranosyl)-5a- cholestane-3β,6β,8,15α,24-pentoI 15-*O*-sulfate(24S)- 24-O-(3-*O*-methyl-β-d-xylopyranosyl)-5a- cholestane-3β,6β,8,15α,24-pentoI 15-*O*-sulfate(24S)- 24-O-(3-*O*-methyl-β-d-xylopyranosyl)-5a- cholestane-3β,6β,8,15α,24-pentoI 15-*O*-sulfate coscinasteroside B coscinasteroside B coscinasteroside B (24S)-24-O-(3-*O*-methyl- β-d-xylopyranosyl)-5a- cholestane-3β,6β,8,15α,24-pentoI 15-O-sulfate B, isolated from the ethanolic extract of the starfish *Ogmaster capella* (Müller & Troschel, 1842) (Valvatida) inhabiting the Western Pacific [132], and granulatoside D (compound **49**, Figure 14), isolated from the ethanolic extract of the starfish *Choriaster granulatus* (Lutken, 1869) occurring in the South Pacific Ocean [133] were studied for bioactivities at 0.1 μM concentration. Both steroid glycosides from *O. capella* at non-toxic concentrations effectively inhibited the ROS level in LPS (1.0 μg/mL)-induced for 24 h RAW 264.7 cells, but not in un-stimulated cells. Neither of the compounds affected the NO levels in macrophages elevated by LPS treatment. Moreover, granulatoside D at a dose of 0.1 μM dually affected ROS, increasing its level in peritoneal murine macrophages and decreasing in LPS—pretreated peritoneal macrophages, which suggests that the effect of this compound on ROS generation depends on the initial environment factors. These findings indicate that the steroid glycosides from the species studied may have oxidant-antioxidant balance modulating capacities, but their possible immunomodulating ability needs to be further studied.

**Figure 15:**
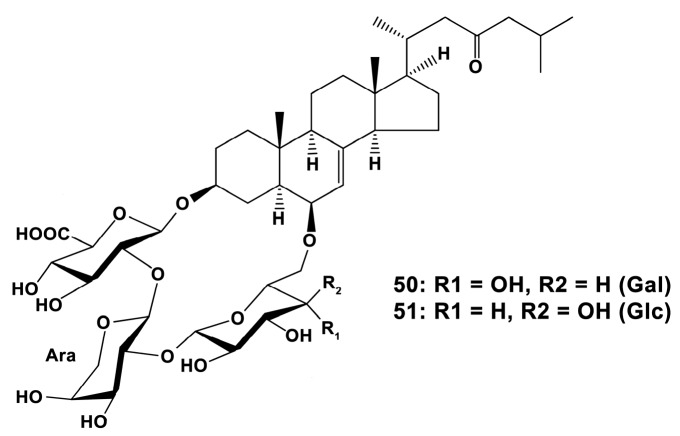
Structures of luzonicoside A (**50**) and luzonicoside D (**51**).

In contrast, another group of steroid glycosides of a rare structure, containing carbohydrate moieties incorporated into a macrocycle, luzonicosides A-E, and a related open carbohydrate chain steroid glycoside luzonicoside F from the tropical and sub-tropical western Indo-Pacific starfish *Echinaster luzonicus* (Gray, 1840) (Spinulosida) increased the ROS level and NO synthesis in RAW 264.7 cells [134]. As these compounds were earlier shown to inhibit also cancer cells [7], these results demonstrate a potential efficacy of luzonicosides, mainly A (**50**) and D (**51**) (Figure 15), against cancer development, and also of stimulating the macrophage to transform phenotype towards the M1 (“anti-cancer”) type.

**Figure 16:**
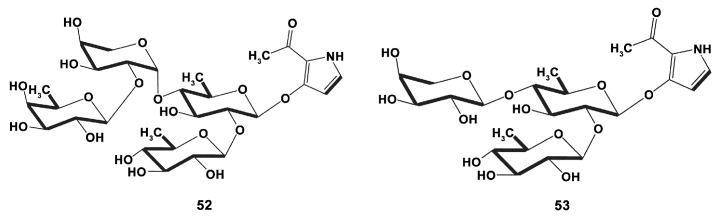
Structure of the plancipyrrosides A (**52**) and B (**53**).

Pyrrole oligoglycosides are also rarely found compounds. New plancipyrrosides (Figure 16) A (**52**) and B (**53**) from the methanol extract of the Vietnamese starfish *Acanthaster planci* (Linnaeus, 1758) (Valvatida) were tested in RAW264.7 cells and shown to have different activity against increased NO production in LPS (1 μg/mL)-stimulated cells after 24 h incubation [135]. Plancipyrroside B (**53**) exhibited a higher inhibitory effect with IC_50_ of 5.94 ± 0.34 µM and may be considered potent for further studies of their immunomodulating properties.

#### 2.5.3. Echinoidea

**Figure 17:**
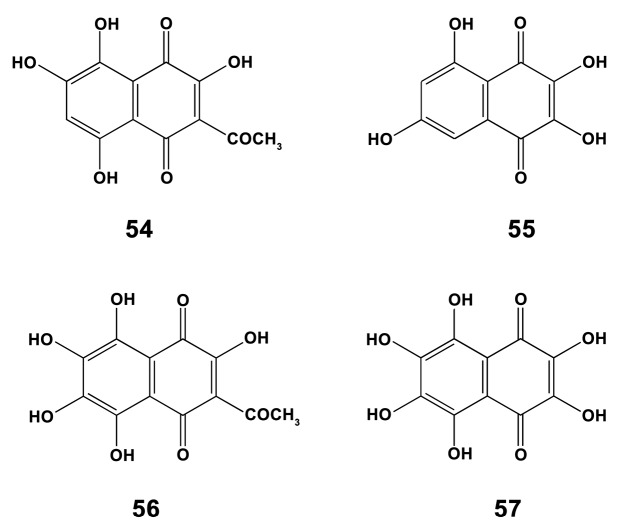
Structure of spinochromes A (**54**), B (**55**), C (**56**), and E (**57**).

Sea urchins contain unique compounds like polyhydroxynaphthoquinones involved in the sea urchin’s pigmentation (spinochromes or echinochromes) [136]. J774 macrophages were used in research on the bioactivity of spinochromes (compounds **54**–**57** in Figure 17) isolated from four common regular sea urchins collected in the Indian Ocean: *Echinometra mathaei* (Blainville, 1825), *Diadema savignyi* (Audouin, 1809) *Tripneustes gratilla* (Linnaeus, 1758) and *Toxopneustes pileolus* (Lamarck, 1816). In LPS (10 ng/mL)-induced macrophages, each of the spinochromes caused an increase in TNF-α production after 6 h incubation while no effect was found without LPS stimulation. One of the possible explanations of such synergistic effect of spinochromes and LPS may be related to a short period of cell incubation, when LPS alone or each of the compounds are not able to induce the massive signaling, and triggering the process of TNF-α production after the multiplying signaling with spinochromes. In any case, spinochromes may be perspective to be used for activation macrophages towards the M1-type, in accordance with data on the pro-inflammatory role of naphthoquinones in mice [137].

In addition, macrophage-stimulating activity was found for SEP-2, a water-soluble polysaccharide, isolated from *Strongylocentrotus nudus* (A. Agassiz, 1864), inhabiting the Yellow Sea, and preliminary characterized as d-glucan containing a (1→4)-linked d-Glcp backbone with (1→3)-linked d-Glcp side chains. SEP-2 was shown to enhance significantly ROS level, NO production, and inflammatory cytokines secretion (IL-1β, IL-6 and TNF-α) in RAW264.7 cell line [138]. These results suggest that SEP-2 might induce macrophage polarization toward the pro-inflammatory M1 state.

The sea urchins are also a rich source for obtaining antimicrobic peptides. One of them, centrocin 1, was isolated from *S. droebachiensis* (O.F. Müller, 1776) (Echinoida) and is characterized as a heterodimer formed by monomers of 30 (heavy chain) and 12 amino acid residues (light chain) connected by a single disulphide bridge. In this study [139], macrophage-like THP-1 cells were stimulated by adding 0.1 ng/mL LPS, concentrations of which were preliminarily selected based on in vitro experiments to give a close to maximum release of cytokines over a period of 6 h. The centrocin 1-peptide derivatives at nontoxic concentrations significantly reduced the level of the pro-inflammatory cytokine TNF-α in LPS-stimulated cells, and some of the peptides reduced the production of another inflammation marker, IL-6, in LPS-stimulated cells. Thus, these peptides may have a potential for using in promoting macrophage phenotype towards M2 type.

#### 2.5.4. Holothuroidea

**Figure 18:**
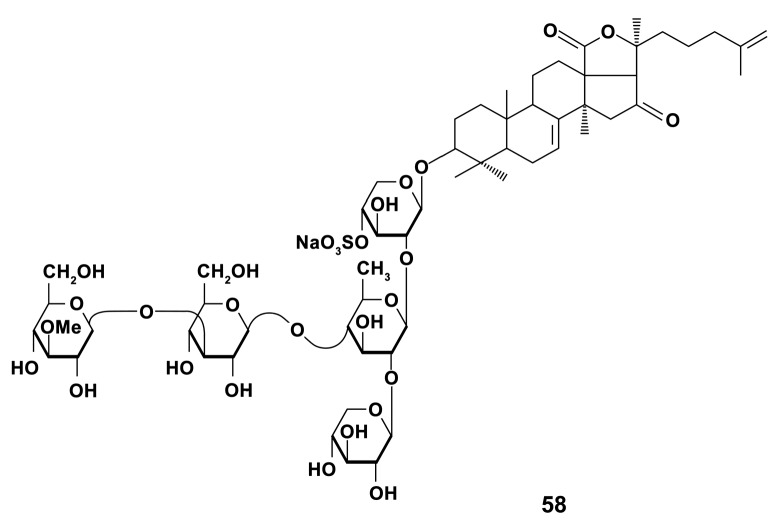
Structure of cucumarioside A_2_-2.

There are about 1500 species of sea cucumbers [140]. Many species are of commercial value [141] and are the main objects of studies for prospective using in pharmaceutical industry. The immunostimulating substances from holothurians of the Far-East Pacific were the most studied. Triterpene glycoside cucumarioside A_2_-2 (A_2_-2) (compound **58**, Figure 18), isolated from the Far Eastern sea cucumber *Cucumaria japonica* (Dendrochirotida), is known to exert significant immunomodulatory effects in vivo [142]. The work of Pislyagin et al. [143] is, apparently, one of the first of those clearly indicating that immunomodulatory activity of marine triterpene glycosides is associated with effects on macrophage polarization in vivo and in vitro. A_2_-2 (1.5 mg/kg) stimulates NO and ROS production in the spleen macrophage in comparison with LPS (1.5 mg/kg) activity that indicates an activation of macrophages towards classical phenotype. Studies on RAW 264.7 macrophages treated with LPS (1 μg/mL) and IFN-γ (20 ng/mL) for M1 polarization or IL-4 (20 ng/mL) for M2 polarization showed that A_2_-2 stimulated polarization through the M1-type way.

**Figure 19:**
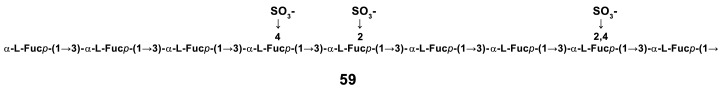
The proposed structure of the SF_3_ fraction from *A. japonicus.*

A number of studies concerned the bioactivity of sulfated fucans of sea cucumbers. The water soluble protein-sulfated fucan (PSF) complex isolated from the body wall of *Apostichopus japonicus* (Selenka, 1867) (old species name is *Stichopus japonicus*) (Synallactida) and four its purified fractions (F_1_, F_2_, F_3_ and F_4_) mostly consisted of neutral sugars, proteins, and sulfates in various proportions, and fucose was their major monosaccharide. A different concentration of samples (2, 5, and 10 μg/mL) induced significant expression of NO, COX-2 and release of pro-inflammatory cytokines IL-1β, IL-6, TNF-α, and IL-12 compared to LPS (1–2 μg/mL), which was used as a positive control in RAW264.7 cells after 18 h of incubation [144]. The enhanced expression of markers of M1 macrophages in cells exposed to fractions displays that fractions could influence classical activation of macrophages. In these studies, the main backbone of highly stimulating markers expression F_3_ fraction (**59**) was (1→3)-α-l-linked fucosyl residue with sulfation at C-2 and/or C-4 (Figure 19). Earlier, a pivotal role of sulfates and M. w. of compounds in activating RAW 264.7 cells was established [145].

**Figure 20:**
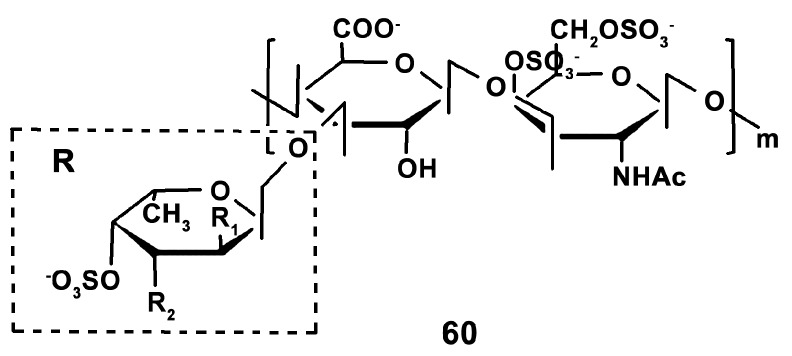
Hypothetical structure for fucose branches stretching from a core chondroitin sulfate unit of intact glycosaminoglycan from *A*. *japonicus* AHG-E.

In addition, the immunostimulating effects of glycosaminoglycan (AHG) (compound **60**) from *Apostichopus japonicus* (Selenka, 1867) (Synallactida) (Figure 20) in cyclophosphamide-induced immunosuppression was studied with using a broad set of markers of mice macrophage activation [146]. It was revealed that AHG (10 mg/kg) enhanced the expression of NO, TNF-α, IL-6, IL-1β, IL-18, and related mRNA, which are the markers of macrophage polarization towards the M1-type [11]. The mechanisms of immunostimulation were shown to be realized through the phosphorylation of MAPK and NF-κB. Apparently, glycosaminoglycan stimulates the classical way of macrophage activation.

In contrast, ethyl acetate solvent fraction (phenol content of about 20.4 mg/g) of *A. japonicus* at concentrations of 10, 50, and 100 μg/mL significantly inhibited the production of NO and PGE_2_ by inhibiting iNOS and COX-2 at their protein and gene levels, as well as production and the gene transcription of proinflammatory cytokines IL-1β, TNF-α in LPS (1 μg/mL) stimulated RAW264.7 cells [147]. The responsible molecular signaling for these effects was found to be through suppression of the phosphorylation of MAPK molecules; ERK and p38 MAPK [148]. These findings, apparently, indicate that phenols of the sea cucumber are able to reverse the M1-type polarization of macrophages.

For screening new metabolites, as well for understanding some mechanisms of macrophage polarization, more simple models may be useful. Previously, P1 and P2 types of phagocytes of the Far-Eastern sea cucumber *Eupentacta fraudatrix* (Dendrochirotida) were shown to be phenotypically distinct and similar to M1 and M2 macrophages, respectively [21]. Using phagocytes as a model for studying a new extract of Far-Eastern holothurians [149], rich with carotenoids and n-3 PUFA as the main biocompounds, both the extract (0.01, 0.1 and 1 μg/mL) and dexamethasone as positive control (0.1, 1 and 100 μM) were shown to downregulate the level of IL-1α-like substances in P2 phagocytes after 24 h incubation in a concentration-dependent manner. After 48 h, the changes in the levels of pro-inflammatory cytokine-like substances induced by both preparations in P1 cells were opposite to those induced in P2 type phagocytes. This fact indicates that inhibition of pro-inflammatory cytokines is involved in the mechanism of action of the extract and corresponds to known wound-healing activity of the extract [150]. Further studies are needed for confirmation of the potent immunomodulating activity of the extract and the mechanisms involved.

## 3. Conclusions

The new paradigm of polarization of macrophages via two distinct ways has founded the understanding of impaired switching between M1 and M2 phenotypes of macrophages as triggering mechanism of development of pathological states and diseases. As a result, correction of M1/M2 balance has become a new therapeutic target, as well as the modern pharmacological trend.

Based predominantly on the evaluation of changes in NO and COX-2 protein levels, analysis of the numerous data on the effects of marine extracts and compounds of different chemical structure in LPS-stimulated macrophages highlighted the fact that soft corals are rich in potentially active substances modulating macrophage polarization towards M2 (anti-inflammatory) type. The most frequent potent activity was found for cembrane-based and eunicellin-based diterpenoids, sterols, ceramides, and cerebrosides.

Studies on pure compounds from Porifera were rarer, but the recently studied extracts were shown to promote M2-way of macrophage polarization with the use of a broad set of the markers, including interleukins and cell surface markers.

In addition, several extracts from Molluscs of different orders (Aplysiida, Mytilida, Cephalaspidea, Ostreida) were argued to be promising for promotion of M2-type polarization. These extracts were rich with carotenoids as well as with n-3 PUFA and n-6 PUFA, which contributed to the extract’s activities. However, compound 1-*O*-alkyl-sn-glycerols from the Oegopsida was shown to stimulate macrophages towards M1 phenotype.

Carotenoid, EPA, and DHA-rich lipids from Crustaceans (krills and shrimps) have shown the promising activity regarding M2-polarization promotion. Different derivatives of polysaccharide chitin have been revealed to activate macrophage polarization through both M1 and M2 ways.

Compounds from tissues of Echinoderms (Holothuroidea, Echinoidea, Asteroidea, Crinoidea) were more often studied with a focus on their macrophage-polarizing properties. The larger proportion of those compounds studied had M1 macrophage promoting abilities (triterpene glycoside cucumarioside A_2_-2, protein-sulfated fucan complex, glycosaminoglycans from sea cucumbers; polyhydroxynaphthoquinones (echinochromes), water-soluble polysaccharide SEP-2 from sea urchins; steroid glycosides luzonicosides from sea stars).

Obviously, the chemical composition and structure of marine substances influence the way and intensity of their effects on macrophage polarization, but exact mechanisms of such dependence are little studied. However, the predominant capacity of more hydrophilic compounds, mostly from echinoderm tissues, and also from squid tissues, compared to that of the hydrophobic molecules produced by other invertebrates, to stimulate classical way of macrophage activation suggests that hydrophilic properties are important for the compound binding to different surface receptors of macrophages and for subsequent signaling cascade. This may have an implication in further constructing new drugs.

On the other hand, most of the reports cover the compounds that promote the reduction in classic polarization of macrophages. This apparently results from the screening character of the studies aimed to establish the anti-inflammatory properties of molecules and using the corresponding stimuli to promote the M1-way macrophage activation in control cells. Nevertheless, the rare reports on the investigation of marine molecule effects in macrophages stimulated toward the M2 phenotype extends the understanding of the mechanisms of macrophage polarization shift, and indicate the importance of the evaluation of the effects of compounds on both types of macrophages.

On the whole, the species of different phyla of marine invertebrates are a promising source to obtain drugs that affect the polarization of macrophages towards both the M2 and the M1 types. Further fundamental research of the mechanisms of the effects of marine compounds on macrophage polarization switching is particularly important for creating new medicines with strong anti-inflammatory or immunostimulating properties.
